# What's in a Name? Interlocutors Dynamically Update Expectations about Shared Names

**DOI:** 10.3389/fpsyg.2016.00212

**Published:** 2016-02-26

**Authors:** Whitney M. Gegg-Harrison, Michael K. Tanenhaus

**Affiliations:** ^1^Writing, Speaking, and Argument Program, University of RochesterRochester, NY, USA; ^2^Department of Brain and Cognitive Sciences, University of RochesterRochester, NY, USA

**Keywords:** common ground, reference, perspective-taking, belief-updating, conversation

## Abstract

In order to refer using a name, speakers must believe that their addressee knows about the link between the name and the intended referent. In cases where speakers and addressees learned a subset of names together, speakers are adept at using only the names their partner knows. But speakers do not always share such learning experience with their conversational partners. In these situations, what information guides speakers' choice of referring expression? A speaker who is uncertain about a names' common ground (CG) status often uses a name and description together. This N+D form allows speakers to demonstrate knowledge of a name, and could provide, even in the absence of miscommunication, useful evidence to the *addressee* regarding the *speaker*'s knowledge. In cases where knowledge of one name is associated with knowledge of *other* names, this could provide indirect evidence regarding knowledge of other names that could support generalizations used to update beliefs about CG. Using Bayesian approaches to language processing as a guiding framework, we predict that interlocutors can use their partner's choice of referring expression, in particular their use of an N+D form, to generate more accurate beliefs regarding their partner's knowledge of other names. In Experiment 1, we find that domain experts are able to use their partner's referring expression choices to generate more accurate estimates of CG. In Experiment 2, we find that interlocutors are able to infer from a partner's use of an N+D form which other names that partner is likely to know or not know. Our results suggest that interlocutors can use the information conveyed in their partner's choice of referring expression to make generalizations that contribute to more accurate beliefs about what is shared with their partner, and further, that models of CG for reference need to account not just for the status of *referents*, but the status of *means of referring* to those referents.

## 1. Introduction

One of the most basic things we do with language is refer to things in the world. When we say something like, “Can you bring me the ball?,” we are using the definite noun phrase *the ball* to refer to a particular ball in the world, and we're hoping that our addressee will be able to execute the requested action. In order to successfully refer, speakers must choose a referring expression that can be understood by their addressee(s), and this requires speakers to take into account what is in *common ground* (CG): the knowledge that is shared between conversational partners. This is particularly relevant when we consider the many choices for definite referring expressions—we can refer via a possessive, as in *my ball*, a definite description, as in *the ball with smudges on it*, a pronoun, as in *it*, or a proper name, as in *Wilson* - each of which assumes a different knowledge and attentional state on the part of the addressee (Grosz and Sidner, 1986; Gundel et al., 1993; Roberts, 2004), and can reflect the status of the referent in the preceding discourse (e.g., Ariel, 1990; Lambrecht, 1994). For proper names in particular, speakers need to know not just that the referent itself is in CG, but that their partner knows it *by that name*. What sources of information are available and used as the basis for our beliefs about what someone else knows, such that we could use this information in guiding our choices about what to say?

A major debate within the literature on CG focuses on the presumed computational complexity of generating and using representations of our partner's knowledge during language production or comprehension. An influential account from Clark and Marshall (1978, 1981) suggested that interlocutors could rely on elaborate “reference diaries” in memory, which enable them to find instances of “triple co-presence” between the speaker, addressee, and referent, and thus safely assume that a particular referent is mutually known. However, these rich representations strike many as psychologically implausible. Some alternate proposals hold that we are by nature egocentric, and instead of using information about their partner's knowledge or perspective, interlocutors use a heuristic: they assume that their own perspective or knowledge can serve as a proxy for what their interlocutor will know (e.g., Keysar et al., 2000; Wu and Keysar, 2007). Under these accounts, taking a partner's perspective into account requires effortful adjustment and monitoring processes, after something has gone awry. Several studies do suggest we are susceptible to making errors about what others know (e.g., Fussell and Krauss, 1992; Epley et al., 2004; Birch and Bloom, 2007), and that in particular, we fall victim to a “Curse of Knowledge” effect: we systematically assume that people know what we know.

But other researchers, such as Brown-Schmidt and Hanna (2011), argue that CG information is one of many partial constraints on language processing and production, and that information about ground status can, at least in cases where the cues to it are strong enough, influence language processing and production from the earliest moments. Brown-Schmidt and Hanna (2011) give an excellent overview of this debate, and point to a number of studies in which there is solid evidence for the use of CG as a constraint in both comprehension and production (e.g., Nadig and Sedivy, 2002; Clark and Krych, 2004; Brown-Schmidt et al., 2008; Brennan and Hanna, 2009; Brown-Schmidt, 2012). Studies from Heller et al. (2012) and Gorman et al. (2013) on referring expression choice, which we summarize in the following section, also lend support to this view of CG. The experiments we present in this paper build off this earlier work to ask whether interlocutors are capable of using the information conveyed by their partner's choice of referring expression to update their beliefs about what their partner knows.

### 1.1. Previous work using names to study CG-use in production

Proper names are arbitrary labels that can only be understood if the addressee knows the link between the label and the referent, and as such, they are ideal tools for exploring the use of CG in production. By teaching overlapping but non-identical sets of names to partners, it is possible to set up situations in which a name is either *privileged* (known only to one of the partners) or *shared* (known to both partners); in order to refer felicitously, speakers should only use those names which they know to be shared. Wu and Keysar (2007) used this paradigm to test their hypothesis that speakers use an “information overlap” heuristic to estimate common ground; they argued that instead of tracking the ground status of individual items, speakers instead rely on their estimate of the overall overlap between their own knowledge and their partner's. Indeed, in the *high overlap* cases (where speakers learned most names with their partner, and only a few were learned alone), they found that speakers used more privileged names than in the *low overlap* cases (where only a few of the names were shared).

However, in their replication of the Wu and Keysar (2007) study, Heller et al. (2012) found that in those cases where speakers used names for privileged items, these names were almost exclusively uttered along with a description, in what they (following Isaacs and Clark, 1987) call the “Name+Description” (N+D) form. Speakers included information that was necessary for their addressees to successfully identify the referent; evidence from additional studies suggested that these descriptions were not simply added as a repair or as a result of miscommunication, but were planned as part of the utterance from the beginning. This suggests that speakers are quite sensitive to the knowledge of their addressees, and skilled at tracking which names are shared, and which are privileged, when the basis for the shared knowledge is shared learning experience. The Name-Alone (N) form is reserved for those items which the speaker believes to be shared, and the N+D form reflects either a belief that that the item is privileged, or a lack of certainty about the item's ground status. But why use the name at all, when the addressee does not know it? Heller et al. (2012) suggested that the use of the N+D form may reflect a teaching strategy; if the speaker believed that they may need to refer to the item on subsequent turns, then it makes sense to use the name along with a description, rather than just a description, in order to “teach” the name to the partner. However, Gorman et al. (2013) explicitly disincentivized teaching by informing participants that they would only see each item once, and found that instead of decreasing their use of the N+D form for privileged items, speakers increased it; post-test debriefings further suggested that these speakers did not believe they were teaching names to their addressee. Though this indicates that speakers were not strategically *teaching* the names to their addressee via the N+D form, this does not mean that addressees would have been incapable of *learning* something from the speakers' use of the N+D form; we will return to this possibility shortly.

Building off the work of Heller et al. (2012), we conducted a series of studies (described in Gorman et al., 2013) exploring the memory representations that support CG use during language production. We based our approach on the framework presented by Horton and Gerrig (2005a,b), who propose that information about CG is represented as a by-product of ordinary memory processes, which contain context-specific episodic traces. Results from the spoken word recognition literature suggest that people might indeed have automatic access to speaker-specific episodic traces for names (Goldinger, 1998; Creel et al., 2008; Creel and Tumlin, 2011). Work on lexical precedents from Metzing and Brennan (2003) and Brown-Schmidt (2009) also demonstrates that addressees can use speaker-specific information when comprehending referring expressions (but cf Kronmüller and Barr, 2015). In short, it seems the representations upon which language use depends (e.g., word representations) already encode speaker-specific information. This might explain how such purportedly rich CG representations as the ones described by Clark and Marshall (1978, 1981) could be used during real time conversation. Our conversational partner and everything about the context in which we are speaking to them serve as cues that make associated information more accessible in memory. One major question, then, is precisely what kind of information needs to be accessible in memory. When we decide we want to refer to something, it seems we need information about whether that referent is “shared” with our addressee (to support what Horton and Gerrig, 2005a would call “commonality assessment”), but also about whether a particular *means of referring* to that referent is likewise “shared” (to support what they would call “message formation”). That is, rather than “triple co-presence,” it seems we need evidence for a sort of “quadruple co-presence”: evidence that we, our addressee, the referent, and the means of referring to it have all been “co-present.” Our work has been aimed at probing the factors that can support inferences regarding the status of particular means of referring to particular referents with a particular conversational partner.

In the studies reported in Gorman et al. (2013), participants learned novel names for novel creatures during the training phase, then interacted during the testing phase in a referential communication game using those creatures. One participant was named the Director, and either learned shared names alongside their partner (the *together* condition), or learned them separately but were told that their partner learned those same names (the *alone* condition). In both conditions, the Director went on to learn a set of privileged names that were not learned by their partner. These studies found support for the Horton and Gerrig (2005a,b) claim that shared experience should enable the development of episodic memory traces linking the speaker, the addressee, and the referents, and thus support the use of CG during production: Directors were far better at avoiding the use of the N form for privileged items in the *together* condition than in the *alone* condition, where such episodic memory traces would not be present. However, even in the *alone* condition, Directors were still much more likely to use the N form for shared items than for privileged items; even without the benefit of shared experience, Directors were still able to use what they had been told about their partner's knowledge, though not nearly as successfully as when the shared knowledge was established through shared experience.

Interestingly, Directors in the *alone* condition were also more likely to use the N+D form for privileged names than Directors in the *together* condition, suggesting that use of the N+D form may reflect greater uncertainty about the ground status of items. One possibility is that the collaborative learning in the *together* condition aided speakers because it provided better context cues to distinguish between shared and privileged information, not just because of partner-specific episodic memory cues. For the Directors in the *alone* condition, almost nothing distinguishes shared and privileged names in memory, since both are learned in isolation. In contrast, in the *together* condition, many pairs collaboratively created memory cues to help remember the names, and the context in which shared names were learned (interacting with another person and the experimenter) and the context in which privileged names were learned (sitting alone with the experimenter) were quite different. As such, another study reported in Gorman et al. (2013) aimed to explore whether the relevant memory cues depend on *partner-specific* shared learning. A *third-party* condition was introduced, in which the Director learned shared names together with a partner, but this partner was not the same partner with whom they would interact in the referential communication task; the Director was simply told that their new partner had learned the same names as their earlier partner. Thus, the shared names were still established via shared experience, but that experience then needed to be generalized to the new partner. It was found that in the *third-party* condition, Directors did nearly as well at avoiding the use of the N form for privileged items as Directors in the *together* condition, but were far more likely to use the N+D form for both shared and privileged items than Directors from either of the other conditions. Directors in the *third-party* condition were able to generalize their shared experience with a third party to their new partner, but were again left with greater uncertainty about the ground status of individual items.

While partner-specific episodic memory traces may be a powerful influence on speakers' ability to use information about the ground status of an item and its name, they are clearly not the only information available to speakers; simply being told what another person has learned was enough to generate at least some use of ground information by the speakers, and learning shared vs. privileged names in more distinguishable contexts helps. This raises a question as to what kinds of representations speakers might be creating in order to accurately remember (or generate expectations about) what names are shared with a particular addressee and to use them (relatively) appropriately, and what kinds of information allow speakers to successfully generalize beyond their direct shared experience with a conversational partner. This is a particularly important question to investigate: we often interact with people with whom we do not share experience, but do share knowledge. Thus, we need to be able to go beyond the direct evidence that comes from shared experience—to make inferences about our partner's knowledge, and generalize beyond those inferences. Our results from Gorman et al. (2013) suggest a potential basis for inferences about shared knowledge based on common community membership (Clark and Marshall, 1978, 1981). Speakers are more likely to use the N+D form when they do not share learning experience with their partner; this allows them to use a name without assuming that their partner knows the name. The speakers' use of this name, in turn, could serve as a cue to the addressee that the speaker is indeed a member of the same community, and thus support a generalization: that the speaker knows other names associated with that community. This chain of inferences and generalizations could support more accurate estimates of CG information over the course of conversation—even when there is no miscommunication between interlocutors.

### 1.2. Motivation for current experiments

Recall that the strongest evidence used to support the argument that speakers are egocentric comes from demonstrations of the “Curse of Knowledge”: the seemingly irrational belief that others know what we know. But is this really so irrational? Consider the task facing speakers interacting with a partner with whom they don't share learning experience: they must adapt to their conversational partner's knowledge in order to refer successfully. Speakers typically interact with partners who are relatively similar to themselves (this is especially true in experiments that involve pairs of college students). In Bayesian terms, people's apparent initial egocentricity may be the result of strong prior expectations that their partner knows what they know (perhaps tempered by the degree to which they see their partner as similar to themselves, and their prior experiences with similar conversational partners and similar conversational contexts). By starting with their own knowledge as a prior estimate of their partner's knowledge, speakers are arguably behaving more rationally than if they used a completely unbiased prior estimate.

Over the course of conversation, interlocutors will be exposed to evidence regarding their partner's knowledge that they could use to update their expectations about what is shared. Recall that Heller et al. (2012) and Gorman et al. (2013) showed that a speaker who is uncertain about a names' CG status often uses the N+D form. This form allows the speaker to demonstrate knowledge of a name without making assumptions regarding whether that name is shared. It thus could provide useful evidence to the *addressee* regarding the speaker's knowledge—and this evidence is available even in the absence of miscommunication. This type of evidence could be particularly useful as a cue to common discourse community membership, if knowledge of one name is associated with knowledge of other names (as in domains where varying levels expertise are associated with use of particular names). Do interlocutors attend to and use this evidence to rationally update their expectations about their partner's knowledge? We explored this hypothesis in two experiments where choice of referring expression could serve as a cue to knowledge of other names. In Experiment 1, we find that domain experts are able to use their partner's referring expression choices to generate more accurate estimates of CG. In Experiment 2, we find that interlocutors are able to infer from a partner's use of an N+D form which other names that partner is likely to know or not know.

## 2. Experiment 1: CG belief-updating in unscripted task-oriented dialog

In Experiment 1, we embedded the task of name learning in the context of a rich “toy” world by creating a role-playing game in which certain levels are always encountered before others, and the participant's choices can make regions of the world (and the information contained there) inaccessible; this makes it possible that a speaker displaying knowledge of one name can implicate that they also have knowledge of the names learned prior to that one in the game. We hypothesized that domain experts could use their partner's referring expression choices to update their beliefs regarding their partner's domain-specific knowledge. We asked the following questions: would Expert's initial beliefs about shared knowledge reflect partner-specific information when available? Could the partner's referring expression choices provide a useful cue to the set of names known by that partner? Would Experts adjust their beliefs about partner knowledge on the basis of their partner's referring expression choices? And could Experts generalize, inferring that one name is known given a display of knowledge of another name?

### 2.1. Methods

#### 2.1.1. Participants

Two native English speakers from the University of Rochester were recruited to serve as Game Experts and were paid hourly rates for their participation. A further 32 native English speakers from the University of Rochester were paid to participate as novice gamers. Four naive participants were brought in for each 2-day experimental session. All participants signed a written consent form which was approved by the Research Subjects Review Board of our institution.

#### 2.1.2. Materials

**Figure 1:**
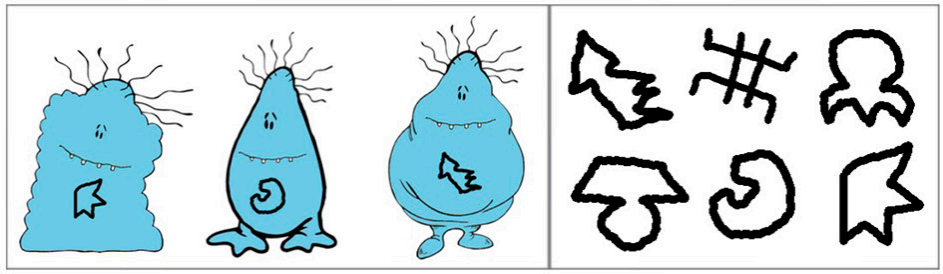
**Experiment 1: example creatures and features**.

Three novel clipart images of “cute monsters” from clipart.com were modified to create nine unique creatures: 3 Wugs, 3 Lorks, and 3 Greps. The individual Wugs, Lorks, and Greps were distinguished by a “feature” designed to resemble a rune, created using the paintbrush in GIMP, drawn on the creature's belly; each feature was assigned an invented name that was one (CVC) syllable in length, and designed to be easily distinguishable from other feature names. Each creature was assigned an invented name (e.g., “Gramperoo”). These creatures were presented over the course of the game. Figure 1 illustrates three sample creatures and all six features used in the game.

#### 2.1.3. Procedure

##### 2.1.3.1. Expert training

In order to create Experts who had full knowledge of everything in the game, each Expert was given a copy of the Game Book (which contained the “story” of the game, along with names and other information about each creature within the game) and specific training. On Day 1, the experimenter took the role of guide, and led the Experts through the game as naive participants. Then the Experts were given 3 weeks to study the Game Book. Experts were quizzed weekly on their memory for the names and other information contained in each level of the game. By the end of training, Experts completed these quizzes with no errors.

##### 2.1.3.2. Day 1

In order to create a situation through which Experts could develop structured shared knowledge with participants, each Expert guided two participants through the role playing game, one at a time. The Expert read the story from the Game Book to the participant, told the participant when to move their game piece on the Game Board, informed the participant of the result of dice rolls and gift choices, and quizzed them on names and other information at the relevant points during the game. Participants played the game twice with the Expert; most did not make it to the final stages of the game.

##### 2.1.3.3. Day 2

A new set of Expert-participant pairings was created: each Expert interacted with one of the participants they'd guided the previous day, and one of the participants the other Expert had guided. Table 1 illustrates the pairings; the order was randomized (Participant A was not always first). This allowed us to explore both partner-specific knowledge and inferences rooted in general expectations for what an unfamiliar game-player might know.

**Table 1:** **Expert-participant pairings**.

**Day 1**		**Day 2**	
**Expert 1:**	**Expert 2:**	**Expert 1:**	**Expert 2:**
Participants A and B	Participants C and D	Participants A and C	Participants B and D

The Experts and Participants completed the following series of tasks on Day 2: a Pre-Test, a Matching Task, a Mid-Test, a SET game, and a Post-Test.

#### 2.1.4. Pre-test

##### 2.1.4.1. Expert

In order to probe the Expert's expectations about their Day 2 partner's knowledge, the Expert was given a worksheet with images of each of the runes and each creature and marked a spot on an 11cm line representing their belief (from “most likely no” to “most likely yes”) regarding the likelihood that the Day 2 partner knew the name. The location of the marks were later measured using a ruler and recorded in a spreadsheet.

##### 2.1.4.2. Participant

In order to establish what the naive participants actually remembered from their Day 1 experience, the naive participants were given a separate worksheet containing images of each of the runes and each creature. They indicated whether they had learned each name, and wrote down the name as they remembered it.

##### 2.1.4.3. Matching Task

The Expert and participant completed a Matching task using cards printed with images of each of the creatures learned in the game, as well as three novel creatures, created using the three family body-shapes, but with rune-markings on the belly that did not correspond to any of the learned characters. The participant and Expert sat in the same room with their backs to one another, so that they could hear each other speaking but could not see the each other's cards. The experimenter placed the cards in a specific order on the participants' table, and then placed the corresponding cards on the Expert's table, in a different specific order. The participant was told to work with the Expert until the cards on the Expert's table were in the same order as the cards on their own table, and the pair was encouraged to converse freely as they worked to accomplish the task. The Matching Task was chosen to provide an opportunity for the naive participant to use the names (and thus provide evidence about their knowledge to the Expert). Their conversation was recorded and transcribed. Transcriptions were later annotated by the experimenter, who marked each reference to a character or rune and tagged it as belonging to one of three categories: Name Alone (N), Name + Description (N+D), or Description Alone (D).

#### 2.1.5. Mid-test

##### 2.1.5.1. Expert

In order to assess changes in the Expert's beliefs regarding their partner's knowledge following the Matching Task, the Expert was given a second copy of the worksheet they completed in the Pre-Test to complete after the Matching task.

##### 2.1.5.2. Participant

The naive participant was given a new worksheet, on which they answered questions regarding the difficulty of the Matching task, and their strategy for referring to creatures they knew and creatures they did not know.

##### 2.1.5.3. SET task

The participant and Expert were seated as in the Matching Task. Each set of cards from the Matching Task were shuffled so that they were in random order; each set was arranged in 3 rows of 4. The Expert and participant were told to work together to form “sets” of cards that shared some common characteristic (e.g., physical appearance, the jobs or territories of the creatures, or the sounds of the creatures' names). The SET task was chosen as a “targeted language game” (Brown-Schmidt and Tanenhaus, 2008; Tanenhaus and Brown-Schmidt, 2008), designed to elicit conversation regarding what each participant knew about the creatures. The Expert and participant took turns choosing two cards from their set of cards; their partner's job was to choose a card from their set of cards that would complete a set with the first two cards that were chosen. They were encouraged to choose their two cards carefully when it was their turn, so that their partner had the best chance of being able to complete a set. The Expert and participant were allowed to tell their partner what characteristic they had in mind for the set, but could not coach their partner on which specific card to use. The Expert and participant were each given one “PASS” to use if they could not complete a set, and were told the goal was to use as many cards as they could before using their PASSes. The conversations and card choices were recorded and transcribed. The transcriptions were later annotated by the experimenter, who marked each reference to a character or rune and tagged it as belonging to one of 3 categories: N, N+D, or D.

#### 2.1.6. Post-test

##### 2.1.6.1. Expert

In order to assess changes in the Expert's beliefs following the SET task, the final worksheet asked Experts to give a final Yes/No judgment regarding their partner's knowledge of names, and also asked about changes in their beliefs about their partner's knowledge from the game over the course of completing the two tasks, and their strategy during the SET task.

##### 2.1.6.2. Participant

The final worksheet asked questions regarding the difficulty of the SET task and their strategies during the SET task, as well as questions about their memory for the names of the creatures and runes, how often they thought they'd used the names, and their strategy for referring to creatures or runes whose names they did not know.

### 2.2. Experiment 1 results

#### 2.2.1. Experts' initial beliefs

We first assessed the Expert's initial beliefs and explored the extent to which these beliefs relate to the knowledge of their Day 2 partner. We converted the Experts' Pre-Test number-line ratings into Yes/No judgments by norming them to a value between 0 and 1, and assigning estimates below 0.5 to No and estimates above 0.5 to Yes. If the partner had answered “no” or gave an incorrect name for an item in the Pre-Test, they were coded as not knowing the name; if they answered “yes” and gave a correct name for an item, they were coded as knowing the name. Experts were correct in their Pre-Test judgments about which names their partner knew and did not know 80% of the time when they were working with the same partner as on Day 1, and 68% of the time when they were working with a different partner than on Day 1, which suggests some use of partner-specific information. Table 2 compares the expectation of the Expert regarding whether their Day 2 partner knew a particular name with the actual knowledge of that partner, as indicated by the partner's Pre-Test responses. Incorrect judgments are bolded. Note that there does appear to be a “Curse of Knowledge” effect in the Experts' response patterns, particularly when the Expert is working with a different partner on Day 2 than on Day 1: Experts assume their partner knows a name when it is not actually known 27.6% of the time when working with the partner, and 42.7% of the time when working with a different Day 2 partner.

**Table 2:** **Expert's Pre-Test judgments of what Day 2 Partner knows and does not know, compared to the Partner's actual knowledge (based on Pre-Test)**.

	**Knows (%)**	**Doesn't Know (%)**
**SAME PARTNER**		
Expert expects Known (33.8% of responses)	72.4	**27.6**
Expert expects Unknown (66.2% of responses)	**15.2**	84.8
**DIFFERENT PARTNER**		
Expert expects Known (15.5% of responses)	57.3	**42.7**
Expert expects Unknown (84.5% of responses)	**28.6**	71.4

However, Experts' basis for their beliefs regarding partner knowledge is more likely to be the experience in the game itself on Day 1, during which their partners may have learned names that were subsequently forgotten by Day 2, and thus in Table 3, we present the percentages of correct and incorrect judgments about partner knowledge broken down by what their partner actually learned, rather than what they report remembering on Day 2; note the dramatic reduction in incorrect judgments about knowledge status for items that the Expert expects to be known (from 27.6 to 2% for the same partners, and from 42.7 to 11.7% for different partners).

**Table 3:** **Expert's Pre-Test judgments of what Day 2 Partner knows and does not know, compared to the names Partner actually learned on Day 1 (Game Experience)**.

	**Learned (%)**	**Didn't learn (%)**
**SAME PARTNER**		
Expert expects Known (33.8% of responses)	98	**2**
Expert expects Unknown (66.2% of responses)	**49.4**	50.6
**DIFFERENT PARTNER**		
Expert expects Known (15.5% of responses)	88.3	**11.7**
Expert expects Unknown (84.5% of responses)	**48.2**	51

To test whether Expert's judgements reflect parter-specific information, we modeled the accuracy (based on their partner's Day 2 Pre-Test knowledge) of Experts' Pre-Test Yes/No judgments about whether a particular item was known using a mixed effects logistic regression model with the partner's status (same as Day 1 or different) as a fixed effect, along with random effects for the partner, Expert, and item. We found a significant main effect of the partner's status, such that Experts were more likely to be accurate in their judgments about whether their partner knew a particular item if their partner was the person they had played the game with on the previous day (β = 0.82, S.E. = 0.39, *p* < 0.05). But when we add Game Experience (whether or not a name was learned by the partner on Day 1) to this model, the partner's Day 2 knowledge is no longer a significant predictor; instead, we find a main effect of Game Experience, such that Experts' ratings of the likelihood that their partner knows a name are significantly higher when that name was learned during Day 1 (β = 1.16, S.E. = 0.38, *p* < 0.01), and there is no interaction with partner status, likely due to the fact that most participants had relatively similar performance (and thus learned similar names) during Day 1. Thus, while Experts' judgments regarding the likelihood that their partner knows a name reflect partner-specific information, they are still relatively accurate for new partners, based on their implicit sense of which names were likely to have been learned during the game.

#### 2.2.2. Patterns of referring expression choice during matching task

**Figure 2:**
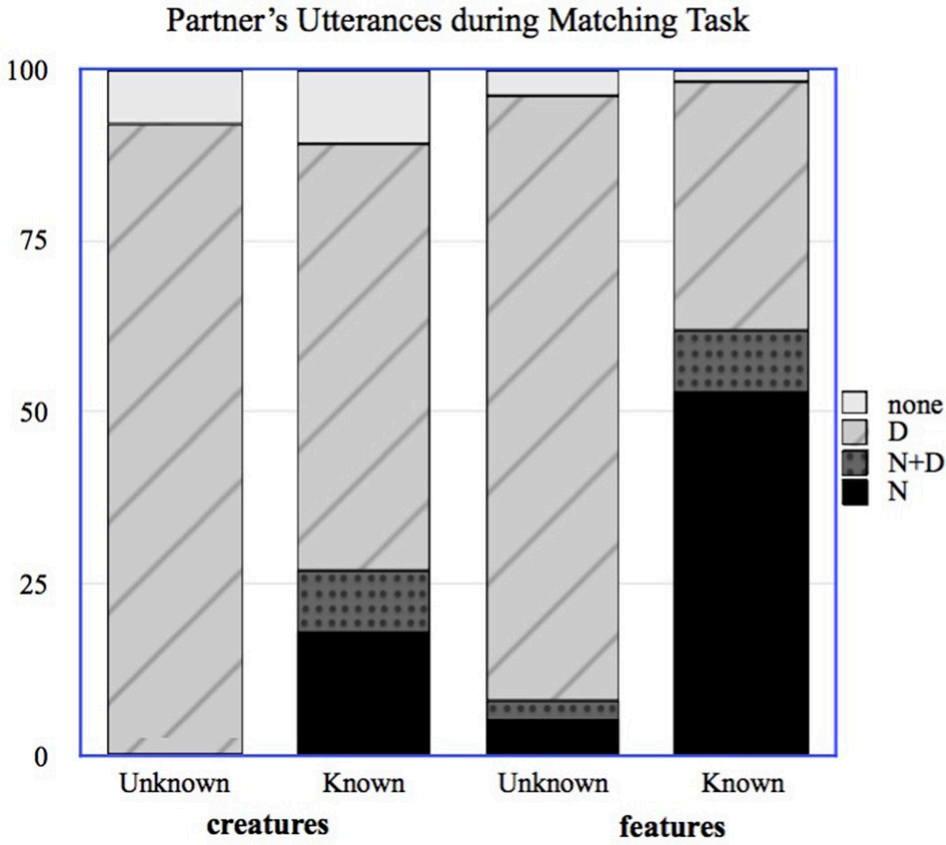
**Distribution of naive participants' utterances for known and unknown creatures and features in the Matching task**.

In order to explore whether the partner's use of referring expressions could have provided a useful cue to the Expert regarding that partner's knowledge, we annotated the transcripts from the Matching task, coding each reference to a named feature or creature as either an N, an N+D, or a D. If a creature was referred to using a description that included the feature name (e.g., “The Wug with the Bor” in reference to the creature *Molgiroo*, a “Wug” family creature with the “Bor” feature), this was coded as an N for the feature and a D for the creature; features could also be described rather than named, as in “The Wug with the thing that looks like a rocket,” and this would be coded as a D for both the feature and the creature. Because of the nature of the matching task, participants were sometimes able to complete the task without referring to all of the creatures; these were coded as “None.” We then recorded the final utterance type from the naive participant referring to that feature or creature (N, D or N+D). This gave us a measure of the evidence provided by the participants' utterances. Figure 2 shows the distribution of the partners' first utterances during the Matching task for known and unknown creatures and features.

For items whose names the partner did know, according to the partner's Pre-Test, the partner used the N form 37% of the time, the N+D form 9% of the time, and the D form 48% of the time. The vast majority of names used by the partner were for features; 81% of the partner's N uses and 53% of the partner's N+D uses were for features rather than for creatures. Note that the partners here are using the N form more often than the N+D form for the names they know; this is likely due to the fact that they are interacting with people whom they know to be Experts, and so can safely refer using only the name. Experts almost never used names during the Matching Task, which was almost certainly driven by the fact that they were playing the role of matcher rather than director.

Note that there are a small percentage of N and N+D uses by the partner for “unknown” items. In all cases where the participant used the N form for an “unknown” item, the participant initially used an incorrect form of the name (and had done so during the Pre-Test as well), and the Expert provided a correction, which the partner then proceeded to use throughout the task, as in the following example:

**Partner:** The next one has a **Rep** on it…

**Expert:** *Do you mean* ***Rab****? The half-star?*

**Partner:** *Oh yes, sorry*, ***Rab****!*

In sum, partners did not use names for all of the items whose names were known to them during the Matching task, but they did use some names. In choosing to use names for particular items, partners may have provided evidence to the Expert regarding their knowledge not only of that item, but of other items that should have been learned alongside it. Likewise, in deciding to use a description for an item, partners may have provided evidence to the Expert that suggested a lack of knowledge of that item's name, evidence that could be misleading if the name is actually known by the partner. Did this evidence contribute to changes in Experts' beliefs about partner knowledge? In the next section, we provide an overview of the changes in Experts' beliefs about what their partner did and did not know, and evaluate the extent to which these changes can be predicted by the evidence provided by the partner during the Matching Task.

#### 2.2.3. Changes in experts' beliefs at mid-test

In order to explore the changes in Experts' beliefs about partner knowledge and the extent to which these changes were driven by the evidence provided in the Matching Task, we first converted the Experts' likelihood estimates from the Mid-Test into Yes/No judgments in the same manner as for the Pre-Test estimates. We find that in the Mid-Test, the difference between Experts' accuracy on Yes/No judgments for same Day 2 partner vs. different Day 2 partner disappear: Experts were correct in their judgments around 79% of the time for both types of partner, meaning that Experts' accuracy for different Day 2 partners improved by 10% over their performance in the Pre-Test.

A summary of the Experts' judgment data from the Mid-Test, broken down by the items the partner did and did not actually know, is given in Table 4.

**Table 4:** **Experts' Mid-Test Judgements relative to Partners' Pre-Test Memory for names**.

	**Knows (%)**	**Doesn't Know (%)**
**SAME PARTNER**		
Expert expects Known (22.3% of responses)	80.7	**19.3**
Expert expects Unknown (77.7% of responses)	**21.1**	78.9
**DIFFERENT PARTNER**		
Expert expects Known (14.6% of responses)	90.6	**9.4**
Expert expects Unknown (85.4% of responses)	**23.2**	76.8

An examination of Table 4 in comparison to Table 2 reveals some interesting changes. Most striking are the improvements in Experts' judgments regarding the knowledge of their partner when their partner was different on Day 2; for different partners, Experts correctly believe things to be known when they are in fact known 90.6% of the time in the Mid-Test, compared to 57.3% of the time in the Pre-Test. The “Curse of Knowledge”-type errors are reduced when working with the same partner, as well: Experts correctly believe things to be known when they are in fact known 80.7% of the time in the Mid-Test compared to 72.4% of the time in the Pre-Test.

In order to assess whether these differences reflected significant improvements in judgements from the Pre-Test to the Mid-Test, we used a mixed effects logistic regression model to predict whether the Yes/No value of the Expert's judgment was correct, with the test from which that judgment came (*Pre-Test* or *Mid-Test*) and the partner status (*same Day 2* or *different Day 2*) as fixed effects and participant number, item name, and Expert name as random slopes and intercepts. We found a significant main effect of judgment type on accuracy, such that judgments that came from the Pre-Test were less likely to be accurate than judgments that came from the Mid-Test (β = −0.58, S.E. = 0.18, *p* < 0.01).

To test whether Experts' Mid-Test judgments were influenced by the evidence provided in the form of their partner's referring expression choices, we used a mixed effects multi-level regression model to predict the Expert's Mid-Test Yes/No judgment for each item, with the referring expression choice of the partner for that item (N, N+D, D, or none) as a fixed effect and participant number, item name, and Expert name as random slopes and intercepts. Experts were significantly more likely to rate an item as “known” when the partner had used the N form to refer to the item (β = 6.45, S.E. = 0.82, *p* < 0.0001) and also when the partner had used an N+D form (β = 3.97, S.E. = 0.86, *p* < 0.0001), and were significantly less likely to rate an item as “known” when the partner had used a description (β = −4.04, S.E. = 0.52, *p* < 0.0001).

We had hoped to test whether Experts would alter their belief about the status of one item based on the evidence provided in relation to another item. However, the patterns of referring expression choice by the partners made that impossible; there were not enough cases where a partner used a name from “later” in the game without also having used one from earlier in the game. There was one striking example, in which the Expert was working with a different partner on Day 2 than he had worked with on Day 1. This partner had made it to the end of the game on Day 1, and during the Matching Task, used names for some, but not all of the creatures she had encountered. She did, however, refer to the final creature from the Day 1 game as “King Floogelor” during the Matching Task, and the Expert reacted with surprise that she knew that name. In this particular case, the Expert dramatically increased his ratings on the Mid-Test (compared to the Pre-Test) for all items. But this was the only example of this type in the dataset. In order to ask specifically about whether the use of one name can lead to generalizations about knowledge of other names, it may be necessary to use partially-scripted games; we present one such approach in Experiment 2.

#### 2.2.4. Patterns of referring expression and set choice during SET task

**Figure 3:**
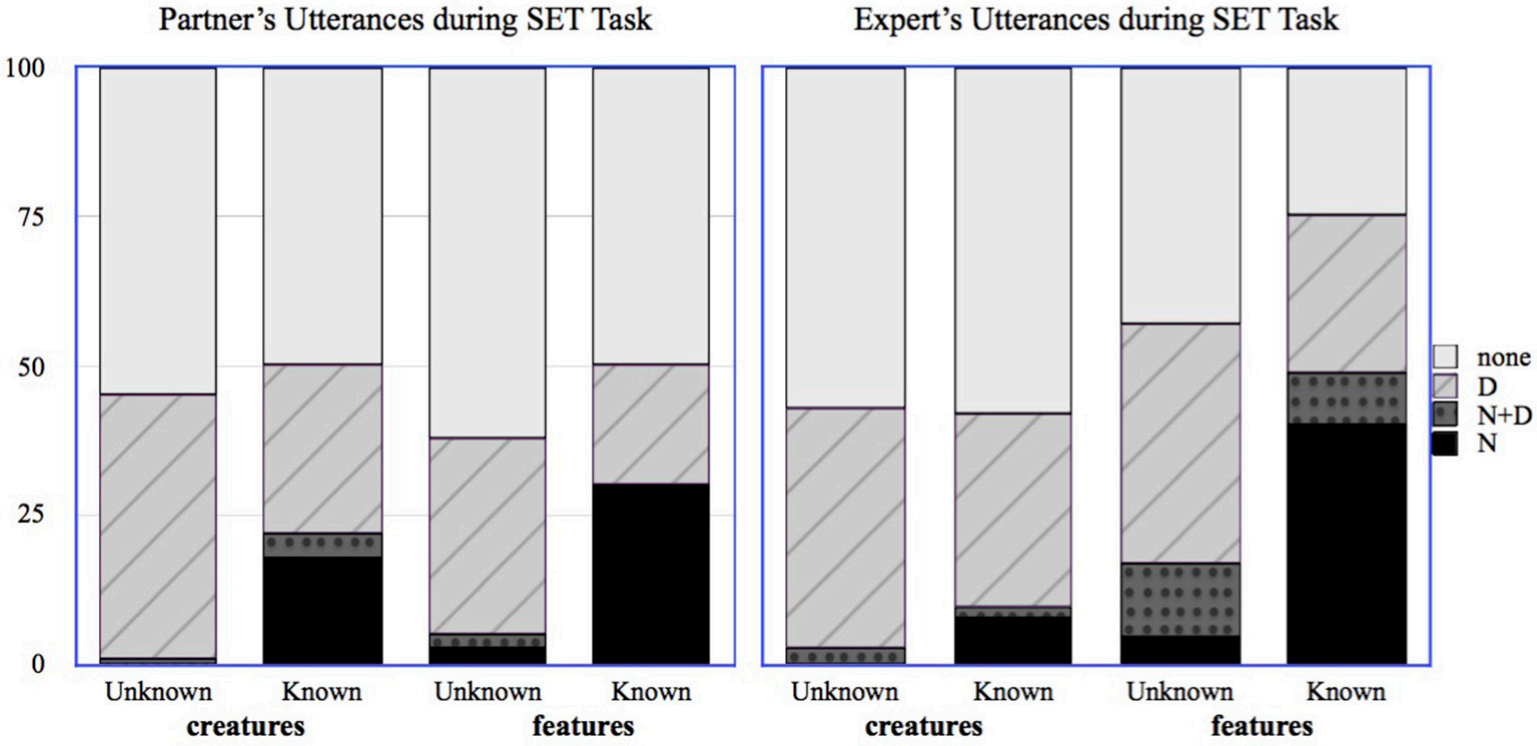
**Distribution of partner's and Expert's utterances for known and unknown creatures and features in the SET task**.

In order to explore whether these changes in beliefs about partner knowledge were reflected in the Expert's referring expression choices during the SET task, we used the same annotation and coding scheme as for the transcripts for the Matching Task, additionally coding the first utterance type from the Expert. Figure 3 gives the distribution of utterance types (N, ND, D, and none) during the SET task for partners and for Experts, respectively.

Unlike the Matching task, Experts used many names during the SET task, restricting their use of names to those items that were actually known by their partner; this was aided by their updated beliefs regarding their partner's knowledge. Models using the Expert's Mid-Test judgments to predict the choice of the N-form in the SET task are a better fit (based on AIC) than those using the Expert's Pre-Test judgments. The connection to the evidence provided by the partner is strong: when we use the form of referring expression chosen by the partner in the Matching Task to predict the form of referring expression chosen by the Expert in the SET Task, we find that Experts are significantly more likely to use the N-form when the partner used the N-form (β = 4.94, S.E. = 0.72, *p* < 0.001) or the N+D form (β = 3.27, S.E. = 0.95, *p* < 0.001) for that item. The handful of instances in which an N form was used for an unknown-to-the-partner item occurred on the final turns of the SET game; at that point it was obvious which card remained, and so the utterance could be understood by the partner even without knowing the name.

### 2.3. Experiment 1 general discussion

Experts' initial beliefs regarding their Day 2 partner's knowledge of names did reflect partner-specific information, as evidenced by the higher accuracy of their Pre-Test judgements when working with the same Day 2 partner as when working with a different Day 2 partner. When working with unfamiliar partners on Day 2, Experts' initial beliefs regarding those partner's knowledge was influenced by the Experts' game-playing experience, and thus still more accurate than might be expected if the Experts' had no basis for forming expectations regarding their partners' knowledge. The partner's referring expression choices during the initial referential communication task did provide useful information regarding the partner's knowledge of names, but partner did not *always* use the N form for names that were known. Experts were able to use the information provided by the form of their partner's referring expressions to generate more accurate beliefs regarding their partner's knowledge of names, which was apparent both in Mid-Test and Post-Test explicit judgements, and in the form of referring expression chosen by the Expert in the final SET task.

There are limitations inherent to the design of this study. Because we used only two Experts, it is difficult to draw general conclusions about what people with expertise do when speaking to those whose knowledge only partly overlaps with their own. Another concern is that by explicitly asking the Experts to make repeated judgments about their partner's likely knowledge, we highlighted the issue of partner knowledge for the Experts in a way that typical conversation does not, and thus made that information more available and salient, which Galati and Brennan (2010) argue is a key factor for finding evidence of CG-use; over the course of the experiment, each Expert was asked to complete 16 Pre-Tests, 16 Mid-Tests, and 16 Post-Tests regarding their partners' knowledge. We can't argue that these tests did not bias the Experts toward more careful attention to their partner's knowledge state, but the results of Experiment 1 do show that it is *possible* for people to attend to the evidence provided by their fellow interlocutor in the form of their choice of referring expression in order to update their beliefs regarding what is and is not shared, and can use this information in deciding how to refer in subsequent conversation.

What Experiment 1 could not reveal is whether interlocutors were capable of generalizing on the basis of their partner's displayed knowledge. In Experiment 2 we explored this possibility using a partially-scripted online game 2 that specifically promotes the use of the N+D form.

## 3. Experiment 2: CG belief-updating in a partially-scripted dialog task

In Experiment 2, we investigated whether a partner's use of a name could lead an interlocutor to generalize about the other knowledge that partner may have. We developed a simplified name-learning game that shared some critical properties with the game in Experiment 1. This game was posted as a “HIT” on Amazon Mechanical Turk, allowing us to obtain data from a larger population. Participants learn the names of creatures while solving increasingly more challenging timed math problems, and then must choose between the Red Path or the Blue Path, and once they choose, they cannot learn names on the alternate path. Thus, participants' knowledge at the end of the game varies, depending both on their ability to solve math problems, and on their choice of path if they make it far enough into the game—a situation that mirrors the one created by the dice rolls and choice points in Experiment 1. Following the name-learning game, participants were asked to take part in a referential communication game with another participant from the name-learning game. This second game player was actually an automated agent, who we will call *AutoTurk*, programmed with particular experiences from the game: in one condition (*RedExpert*), AutoTurk was an expert who made it all the way to the end of the Red Path; in another (*BlueExpert*), AutoTurk made it all the way to the end of the Blue Path; and in the third (*EarlyFailure*), AutoTurk was a poor player, who failed out of the game by solving a math problem incorrectly after learning only two character names. We collected data regarding how participants shifted their expectations regarding partner knowledge during the course of conversation, both via explicit judgments and via their referring expression choices. We hypothesized that upon hearing a partner use a name for an item from the Red Path, participants would be more likely to believe their partner knows other Red Path names, and *less* likely to believe their partner knows Blue Path names.

### 3.1. Methods

#### 3.1.1. Participants

Hundred and twenty naive adult speakers of English volunteered to participate in the study for payment via Amazon Mechanical Turk. Prior to accepting the Mechanical Turk “HIT,” participants gave consent via a digital consent form approved by the Research Subjects Review Board of our institution.

### 3.2. Materials

**Figure 4:**
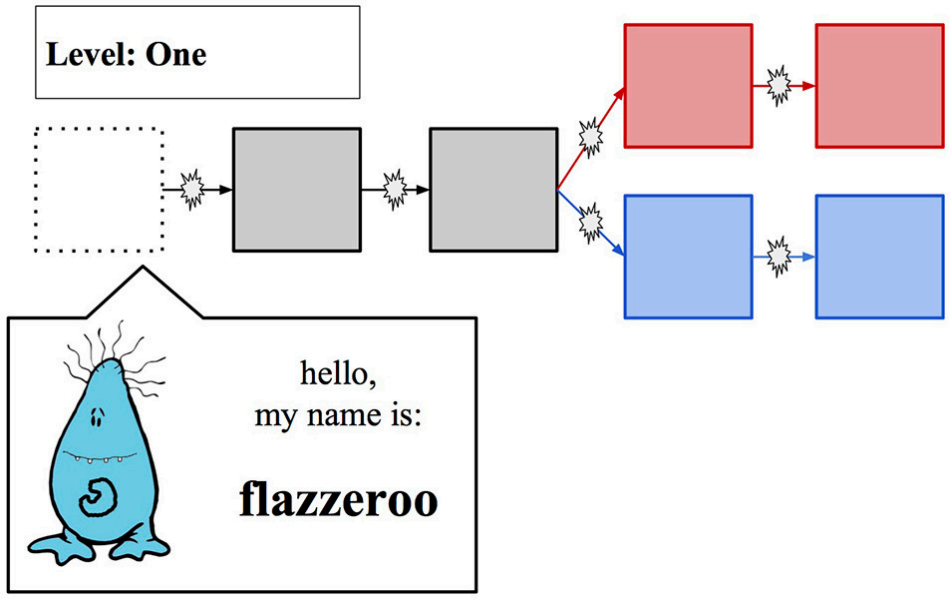
**Game Layout/Introduction of First Creature; each box represents a character whose name could be learned, while each “splat” represents a timed math problem to be solved**. The top path (“Red Path”) had more challenging math problems and a higher payout.

Three novel clipart images of “cute monsters” from clipart.com were modified to create seven unique creatures from three families, each with invented names, as in Experiment 1. These creatures were presented over the course of game, whose layout is depicted in Figure 4. A template was used to generate five unique math problems for each participant. They were asked to solve the problems to progress through the game; each “splat” symbol on the game paths represents a math problem.

### 3.3. Procedure

#### 3.3.0.1. Instructions

The participant, prior to accepting the HIT, read instructions describing the game, its rules and payout structure. Each participant was informed that they would be playing a game that involved two separate stages: learning the names of the cartoon creatures while solving math problems, then playing a matching game with a networked partner who had also completed the first stage. They would acquire points for each correctly learned name and correctly solved math problem during the first stage; these points would carry over into the second stage, where they would acquire points based on how well they solved the matching game with their partner. Participants were also told that if they successfully completed Stage 1 (by making it to the end of either path), they would receive a bonus payout (“Bonus 1”); another bonus payout (“Bonus 2”) was based on the total number points across Stage 1 and Stage 2 combined.

#### 3.3.0.2. Stage 1

The participant was presented with the game screen, and introduced to the first character, as depicted in Figure 4, and told to remember its name. They were then given 8 s to solve a simple addition problem; if successful, they advanced to the next round of the game, and were introduced to another creature. The math problems were designed to have the form X + Y, where X and Y were both single digit numbers for the problems that were solved prior to the path-split. For the last two problems, solved after the path-split, X and Y were both double digit numbers if the participant had chosen the Red Path, while only one of X or Y was a double-digit number if the participant had chosen the Blue Path. Participants were made aware that the choice of the Red Path would entail more difficult math problems, but a higher Bonus 1 payout. If the participant successfully reached the “midpoint” of the game (after learning the third creature), they were given a Mid-test: they had to choose each creatures' name from a list of four possibilities. If successful, they were given a choice between the Red Path and the Blue Path. If the participant successfully learned both names on their chosen path, they were given a final test in which they had to again choose each creatures' name from a list of four possibilities before they could complete the path and advance to the Practice Phase. If the participant incorrectly solved a math problem or failed to successfully complete the Mid-test, their time in Stage 1 ended, and they were advanced into the Practice Phase—this allowed us to create a believable scenario in which a random participant in the game could know anywhere from 0 to 5 names.

#### 3.3.0.3. Practice phase

To ensure that participants remembered the names they had just learned, they were next presented with a series of single creatures in random order, as well as a list of 10 possible names (three were “distractor” names that did not belong to any creature), along with the options “did not learn” and “do not remember.” For each creature, the participant needed to correctly identify its name if it was a creature they had learned, or correctly identify it as an unlearned name if it was a name they had not learned. If the participant chose an incorrect name, or chose “do not remember” for a name they had learned, they were reminded of the creature's name; if they chose any name at all for a creature they had not learned, they were reminded of the fact that they did not learn it. For each creature, the participant needed to correctly identify its name (or status as an unlearned creature) twice before advancing to Stage 2.

#### 3.3.0.4. Stage 2

Participants were told that they were being connected to another Mechanical Turk “worker” who had also participated in Stage 1 of the game, and were shown a “wait” symbol and a progress bar, which changed to a “connection” symbol after a random time lag of between 2 and 90 s; in reality, they were “connected” to *AutoTurk* (the automated agent described earlier). Then, participants were asked to give Yes/No judgments regarding their expectation that their random partner would know each of the seven creatures. Next, participants were told they would be playing a matching game with their partner (who was actually AutoTurk). When it was the participants' turn to be the director, they were shown a single creature; their job was to decide how to refer to that creature so that their partner (actually AutoTurk) could correctly identify it from an array of four creatures.

On director trials, participants were presented with a list of 10 names and a list of 10 descriptions from which they could choose using radio buttons. They were told that if their partner chose the correct creature based on what they said, they would receive 8 points by default; if they used a name along with a description, they'd receive 5 bonus points, and if they used just the name, they'd receive 10 bonus points. If their partner chose the wrong creature based on what they'd said, they'd lose 10 points (Thus, using the name by itself if the partner does not know it would result in losing 10 points instead of gaining 18). The participant took turns with AutoTurk playing the role of director or matcher; when the participant was the matcher, they saw four creatures on the screen, and were presented with the referring expression their partner (actually AutoTurk) had selected: either N, N+D, or D. When the participant was the matcher, they received 10 points for selecting the correct item based on AutoTurk's utterance, and lost 10 points if they chose the wrong one.

Critically, the trials (shown in Table 5) were ordered such that it was possible for the AutoTurk to use either an N+D or a D form for particular creatures, which could then serve as a cue to the participant about whether their partner knew those creatures; in Trial 4 (a Blue Path creature), *BlueExpert* uses an N+D while *RedExpert* uses a D, and in Trial 6 (a Red Path creature), *BlueExpert* uses a D while *RedExpert* uses an N+D. Since participants should only choose the N form for a particular creature if they believed their partner actually knew the name, this allowed us to use subsequent choices between N and N+D for creatures the participant knew as a measure of their beliefs regarding their partner's knowledge.

**Table 5:** **Experiment 2: Stage 2 Trials**.

**Trial**	**Utterance Form/AutoTurk Knowledge**		
	**RedExpert**	**BlueExpert**	**EarlyFailure**
1. Flazzeroo	Participant's Choice		
2. Floogirep	N+D	N+D	D
3. Gramperoo	Participant's Choice		
4. Bampirep (Final Blue)	D	N+D	D
5. Molgirep (Blue)	Participant's Choice		
6. Narpelor (Final Red)	N+D	D	D
7. Trimmelor (Red)	Participant's Choice		
8. Flazzeroo	N	N	N
9. Narpelor (Final Red)	Participant's Choice		
10. Gramperoo	N	N	N

#### 3.3.0.5. Post-Tests

To allow us to track whether participant's explicit judgements of their partner's knowledge shifted as a result of playing the referential communication game, the participant again made Yes/No judgments regarding their belief that the partner they had worked with knew each of the creatures. They were also asked whether they had paid attention to whether their partner used names, and were asked to describe their own strategy for completing the game in Stage 2, and what strategy they thought their partner was using.

### 3.4. Results

#### 3.4.1. Explicit judgments of partner knowledge

One issue worth noting is that 7% of participants, in the Post-Test, gave Yes judgments to all of the creatures; they indicated that they believed the partner they had worked with knew all of the creatures from the Red Path and from the Blue Path, which is impossible. This suggests that at least some participants did not recognize the path split during Stage 1 for what it was, and thus eliminates any expectation that these participants could draw an inference from the fact that the AutoTurk used a name from one of the two paths. Another 6% of participants gave responses that indicated they believed their partner knew creatures from later in the game but not creatures from earlier in the game: this also indicates a lack of attention to (or memory for) the overall structure of the game. These participants were excluded from our analyses.

We first compared participants' judgments regarding AutoTurk's knowledge collected prior to Stage 2 to those collected in the Post-Test. If participants were behaving optimally, then we should expect that when interacting with *RedExpert*, participants' judgments should shift toward Yes for *Trimmelor* and *Narpelor* and toward No for *Molgirep* and *Bampirep*. When interacting with *BlueExpert*, participants' judgments should show the opposite pattern. And finally, when interacting with *EarlyFailure*, participants should show shifts toward No for all of the late-stage creatures. These patterns are indeed present in the data. We used a linear mixed effects regression model to predict whether the change from Stage 1 to Stage 2 judgments would be positive for individual creatures, with the AutoTurk's knowledge status as a fixed effect and the path chosen by the participant as a random effect. For *Trimmelor* (a Red Path creature), we found that participants interacting with *BlueExpert* were significantly less likely to have a positive shift (β = −1.5, S.E. = 0.4, *p* < 0.001), as were participants interacting with *EarlyFailure* (β = −0.41, S.E = 0.17, *p* < 0.05); participants interacting with *RedExpert* were significantly more likely to have a positive shift (β = 0.89, S.E = 0.14, *p* < 0.001). For *Narpelor* (another Red Path creature), we found a similar pattern; participants interacting with *RedExpert* were significantly more likely to have a positive shift (β = 0.81, S.E. = 0.13, *p* < 0.001) while participants interacting with *EarlyFailure* were significantly less likely to have a positive shift (β = −0.3, S.E = 0.14, *p* < 0.05), as were participants interacting with *BlueExpert* (β = −1.09, S.E. = 0.29, *p* < 0.0001). Overall, participants judgments following Stage 2 do reflect the evidence provided by AutoTurk's utterances.

#### 3.4.2. Referring expression choice

**Figure 5:**
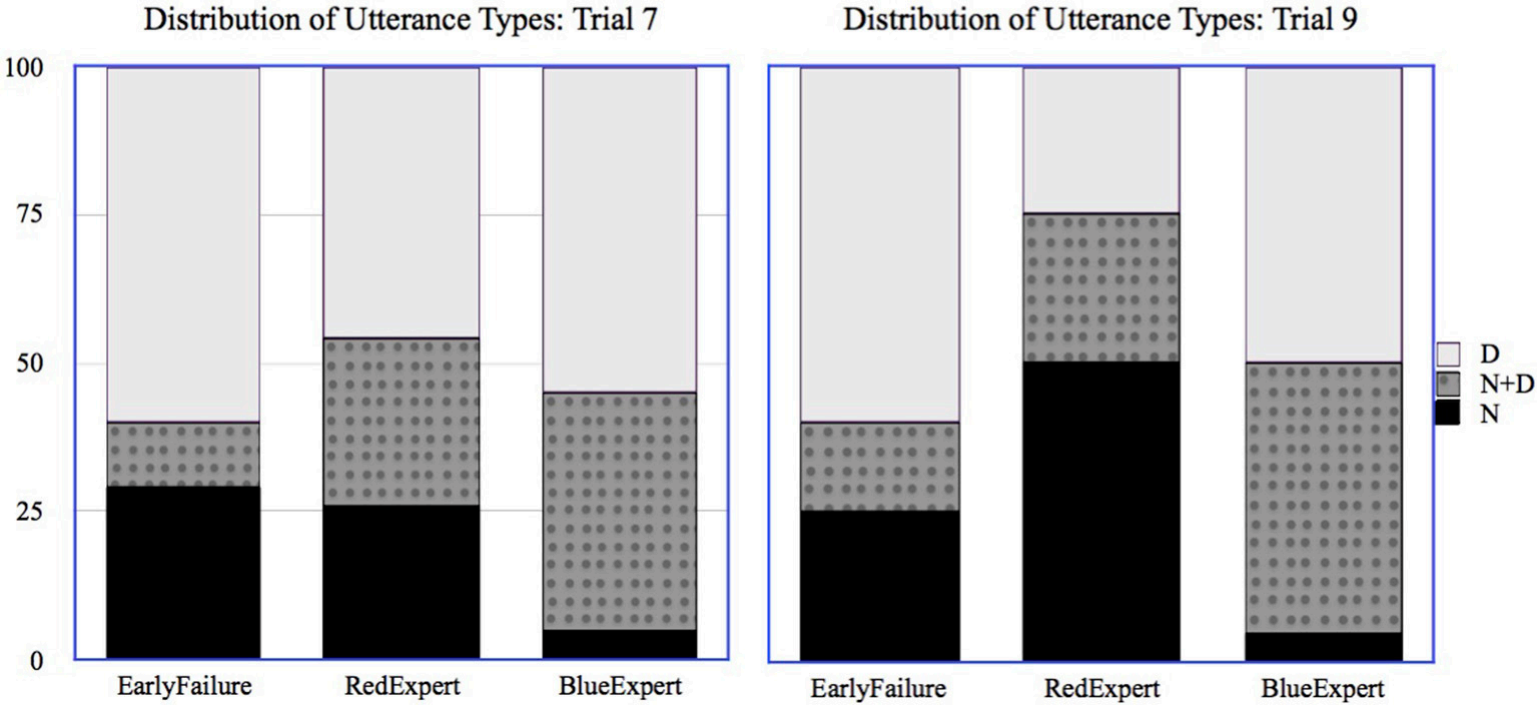
**Distribution of participants' utterance types when interacting with EarlyFailure, RedExpert, or BlueExpert for Trial 7 and Trial 9 (referring to Red Path creature)**.

In order to look at the choice of referring expression in a meaningful way, it was necessary to restrict all analyses to only those individuals who actually knew the relevant name for that trial, and because most participants chose the higher-paying Red Path, we focus our analyses on Trials 7 and 9, in which the target creatures are the two late-stage Red Path creatures. As shown in Table 5, both of these trials followed references to Blue and Red path creatures, and the form of the referring expression used by AutoTurk for those creatures varied depending on AutoTurk's knowledge. Figure 5 shows the distribution of utterance types chosen by participants for *Trimmelor* (Trial 7) and *Narpelor* (Trial 9).

For each trial, we used a mixed effects logistic regression model to predict whether the participant would use the N form for the target creature, with AutoTurk's knowledge as a fixed effect and the path chosen by the participant as a random effect. For Trial 7, we found that participants interacting with *BlueExpert* were significantly less likely to use the N form (β = −2.39, S.E. = 1.15, *p* < 0.01). However, we did not find any significant difference in the likelihood of using the N form between participants interacting with *RedExpert* and *EarlyFailure*; even when participants interact *EarlyFailure*, they appear to be as likely to use the N form as participants who interacted with RedExpert, who has already used a name for a (later) Red Path creature. Thus, the results of Trial 7 only partially support the hypothesis that participants are sensitive to their partners' use of name and adapt their choice of referring expression accordingly. Note, however, that *EarlyFailure* simply uses descriptions all of the time, and that this is not solid evidence of a lack of name knowledge the way that using a name for a creature from the other path is.

For Trial 9, we found that participants interacting with *RedExpert* were significantly more likely to choose the N form (β = 2.7, S.E. = 1.2, *p* < 0.05) than any others, and participants interacting with *BlueExpert* were significantly less likely to choose the N form (β = −2.6, S.E. = 1.3, *p* < 0.05). Thus, at least in the final trial of the experiment, and for an item whose name *RedExpert* had used in an N+D form in a previous trial, participants do seem to take AutoTurk's knowledge into account in their choice to use a name.

We also examined participants' Post-Test descriptions of their own strategy and their beliefs regarding their partner's strategy. Here, we found that most participants focused on the memory-related challenges of the task, commenting on how they kept the creatures' names straight and on how difficult that was. But based on the participants' *post-hoc* reflections on strategy, some individuals believed themselves to be sensitive to the information shared by their partner in the form of their choice of referring expression, and were aiming to make “optimal” referring expression choices based on that information. Many of these participants commented that they thought their partner was using the same strategy, but “doing a better job of it.” But as we noted, the primary focus of participants in their Post-Test comments was on the challenges posed by the memory task; this may suggest that the basis for the inferences we were interested in testing (the overall structure of the game and the names contained within it) was either not recognized or misremembered by some participants.

### 3.5. Experiment 2 discussion

Participants were able to generate more accurate beliefs regarding what their partner knew following interaction with that partner. In the Post-Test, participants changed their beliefs in the expected direction given the knowledge displayed by AutoTurk; thus, even in this simplified game environment, participants are capable of using their partner's utterances to arrive at a more accurate set of beliefs regarding their partner's knowledge.

The participants' referring expression choices also support our generalization hypothesis: in the final trial, we found that participants were significantly more likely to use a name to refer to final Red Path creature if they were interacting with *RedExpert*. Preceding trials paint a somewhat messier picture regarding the relationship between AutoTurk's utterances and the participants' choice of referring expression, particularly in the case of participants interacting with *EarlyFailure*. However, it's worth remembering that in Experiment 1, participants' use of names did not fully reflect their knowledge; participants often used descriptions even when they did, in fact, know the names. In the context of Experiment 2, this means that it is not necessarily valid to make the inference that because *EarlyFailure* used a description, it must not know the name. But we still would have expected participants interacting with *EarlyFailure* to use the N+D form, rather than using names by themselves, given the lack of evidence *for* knowing names. Still, participants interacting with *BlueExpert* were the least likely to use names; it seems that participants could in fact generalize from *BlueExpert*'s use of the N+D form for a Blue Path creature, and infer that *BlueExpert* could not possibly know the name of a Red Path creature.

Given the extent to which the kinds of belief updating we are interested in would depend on both accurate mental representations of the structure of the knowledge domain and attention to the partner's utterances, the combination of Mechanical Turk and novel knowledge may not have been a good one. Though many psychological findings have been successfully replicated using Mechanical Turk (e.g., Munro et al., 2010; Crump et al., 2013), the Mechanical Turk platform by itself does nothing to promote attention to the task unless the creator of the HIT creates incentives in the form of bonuses for work that meets some kind of standard. But in developing the bonus scheme to motivate participants to attend to the task in Experiment 1, we might have been probing participants' gambling behavior, rather than their conversational behavior: would a participant be willing to risk losing 10 points for the chance of gaining 18? What if the point spread were different? And indeed, many participants commented on making this calculation as if it were a bet, when they described their strategy in the Post-Test.

Even more important, though, is the difficulty that participants had remembering the names and the overall structure of the game in Stage 1. Based on their Pre-Test responses, participants struggled to remember which creature was which, and many of them seemed not to remember the order in which creatures were learned (or even that the path split meant that some creatures could not be learned together), and thus it seems unlikely that the kinds of memory associations that would be necessary in order for category-related cues to be useful for referring expression choice would even be present for these participants. These memory and knowledge-structure issues are crucial areas for future work; it seems likely, for example, that a sleep interval may be necessary in order to develop the kinds of concept associations necessary for these inferences (Stickgold and Walker, 2013; Landmann et al., 2014).

## 4. General discussion

Experiments 1 and 2 demonstrate that interlocutors are capable of using information gained over the course of conversation, particularly the information conveyed by the partner's choice of referring expression, to update their expectations regarding what knowledge is shared with their partner, and that these belief updates influence speakers' choice of referring expressions during subsequent conversation. In Experiment 2, we found some evidence for generalization, in that participants interacting with *BlueExpert* were the least likely to use the N form for a subsequent reference to a Red Path creature; the demonstrated knowledge of Blue Path names allowed participants to generalize to a necessary *lack* of knowledge of Red Path names. These findings have important implications for theories of CG use during conversation.

In many theories, the distinction between knowing that *a referent* is in CG (and is thus something to which the speaker could felicitously refer) and knowing that *a particular means of referring to that referent* is likely to be understood by the addressee, seems to be either blurred or non-existent. Yet even in those cases where an object is clearly in CG via a cue like visual co-presence (as in “cubbyhole” studies, e.g., Keysar et al., 2000), the speaker still has to decide what to call it. Even for common nouns, we usually have a choice regarding which to use to refer to a particular item: do we call something a *cassette* or a *tape*? While a number of factors influence this decision (word frequency, the other items in the display, etc), one of these should surely be whether or not the addressee is familiar with the link between a particular expression and the referent. This is especially true for proper names, which are arbitrary labels for a referent, and can only be understood by those who know about the link between the label and the referent. But it seems possible that various kinds of referring expressions could come to serve, under certain circumstances, as context-dependent conventions for referring (in other words, as context-dependent names), and this may be a way of connecting the work presented here to studies of lexical precedents or conceptual pacts (e.g., Metzing and Brennan, 2003; Brown-Schmidt, 2009). Partner-specific expectations relating to the means of referring could also play a role in comprehension, and in pilot work building off Wolter et al. (2011), we are currently exploring whether a speaker's use of a scalar contrast (e.g., *the big candle*) generates an expectation that the contrast item will be called *the small candle* even when the contrast is no longer present, by looking for reduced cohort competition effects with a competitor like *candy*.

We suggest that what is truly needed in an account of CG for definite reference is not triple co-presence of the sort described by Clark and Marshall (1978, 1981), but rather, quadruple co-presence: there must be some record in memory that links the speaker, the addressee, the object, and *a particular means of referring to that object* in order for a speaker to have a reasonable expectations that their use of that means of referring to that object will be understood by their addressee. Community membership can provide a powerful cue to such co-presence; we can often safely assume that certain means of referring to objects will be known by people by virtue of who they are and the communities to which they belong. This does not necessarily require any elaborate representations along the lines of the reference diaries proposed by Clark and Marshall; indeed, if this kind of knowledge can be drawn upon via the associative mechanisms proposed by Horton and Gerrig (2005a,b), it could underlie the kinds of belief-updating we described earlier. If we learn through conversation that our partner is the member of a particular community, this may trigger associations with the kinds of knowledge members of that community have (both in terms of referents and in terms of means of referring to those referents) that could lead to more accurate estimates of CG. And in particular, the evidence that could trigger such associations could come through our partner's use of an N+D form, as suggested by the results of Experiments 1 and 2, since that form enables speakers to display knowledge of a name without making any assumptions about whether it is shared with their addressee.

But not all displays of knowledge are equivalent. Finding out that an interlocutor knows the name *Statue of Liberty* or *guitar* is not particularly informative, as nearly any speaker of English would know those names. But evidence that the speaker knows the name *South Street Seaport* or *viol da gamba* should cause their conversational partner to shift toward believing their fellow interlocutor has expertise with New York City landmarks or Renaissance-era stringed instruments, respectively. If language comprehension is, in part, a process of trying to explain why the speaker said what they said the way that they said it (as in, e.g., Hobbs et al., 1993), then the explanation we seek may be rooted in what we think the speaker knows. Bayesian belief-updating could provide a useful framework for exploring the extent to which interlocutors use evidence to generate such explanations in a rational way, based on the informativity of the evidence provided by their partner. We have ample evidence that during interactive conversation, interlocutors adapt their expectations regarding such things as the likelihood of particular syntactic constructions (e.g., Kleinschmidt et al., 2012; Fine et al., 2013) or of particular phonetic realizations (e.g., Kleinschmidt and Jaeger, 2015), and both of these adaptation phenomena have been successfully explored using Bayesian belief-updating models. Future work in this area should focus on understanding the process by which people generate, update, and generalize beyond their prior expectations regarding partner knowledge; games like the ones used in Experiments 1 and 2 may provide a fruitful paradigm for exploring these processes in more detail, particularly as they relate to the relative informativity of a particular knowledge-display.

The choice of referring expression is a valuable tool for exploring questions about how we use CG. Even in those cases where an object is physically co-present and therefore can be referred to using a definite NP, speakers still must decide what to call it; thus, it is crucial not to think about CG simply in terms of what interlocutors know about what their conversational partner can *see*, but to also consider what interlocutors believe about what their partner *knows*. What's in a name? Evidence about knowledge.

## Author contributions

All authors listed, have made substantial, direct and intellectual contribution to the work, and approved it for publication.

### Conflict of interest statement

The authors declare that the research was conducted in the absence of any commercial or financial relationships that could be construed as a potential conflict of interest.
